# Respiratory Failure due to Possible Donor-Derived* Sporothrix schenckii* Infection in a Lung Transplant Recipient

**DOI:** 10.1155/2015/925718

**Published:** 2015-11-30

**Authors:** Nathan C. Bahr, Katherine Janssen, Joanne Billings, Gabriel Loor, Jaime S. Green

**Affiliations:** ^1^Division of Infectious Disease and International Medicine, Department of Medicine, University of Minnesota, 420 Delaware Street SE, Mayo Mail Code 250, Minneapolis, MN 55455, USA; ^2^Department of Medicine, University of Minnesota, 401 East River Parkway, VCRC 1st Floor, Suite 131, Minneapolis, MN 55455, USA; ^3^Division of Pulmonary, Allergy, Critical Care, and Sleep Medicine, Department of Medicine, University of Minnesota, 420 Delaware Street SE, Mayo Mail Code 276, Minneapolis, MN 55455, USA; ^4^Division of Cardiothoracic Surgery, Department of Surgery, University of Minnesota, 420 Delaware Street SE, Mayo Mail Code 207, Minneapolis, MN 55455, USA

## Abstract

*Background*. De novo and donor-derived invasive fungal infections (IFIs) contribute to morbidity and mortality in solid organ transplant (SOT) recipients. Reporting of donor-derived IFIs (DDIFIs) to the Organ Procurement Transplant Network has been mandated since 2005. Prior to that time no systematic monitoring of DDIFIs occurred in the United States.* Case Presentation*. We report a case of primary graft dysfunction in a 49-year-old male lung transplant recipient with diffuse patchy bilateral infiltrates likely related to pulmonary* Sporothrix schenckii* infection. The organism was isolated from a bronchoalveolar lavage on the second day after transplantation. Clinical and radiographic responses occurred after initiation of amphotericin B lipid formulation.* Conclusion*. We believe that this was likely a donor-derived infection given the early timing of the* Sporothrix* isolation after transplant in a bilateral single lung transplant recipient. This is the first case report of sporotrichosis in a lung transplant recipient. Our patient responded well to amphotericin induction therapy followed by maintenance therapy with itraconazole. The implications of donor-derived fungal infections and* Sporothrix* in transplant recipients are reviewed. Early recognition and management of these fungi are essential in improving outcomes.

## 1. Background

Invasive fungal infections (IFIs) cause significant morbidity among solid organ transplant (SOT) recipients, with mortality ranging from 27 to 41% [1]. The impact of donor-derived infections in SOT is increasingly recognized, with 40% mortality related to donor-derived infections (bacterial, fungal, and parasitic) from one study [2]. When 23 cases of donor-derived invasive fungal infections (DDIFIs) were reviewed by Gomez and Singh in 2013, the median time of onset was 21 days after transplant [3]. Clinical manifestations included vascular complications at the graft site (65%), acute graft dysfunction (43%), unexplained febrile illness (39%), and a graft loss rate of 83% [3]. Risk factors for donor transmission included donors who were transplant recipients themselves or had near-drowning aspiration events, recipients who were transplant tourists, or those whose organ was contaminated during the transplant process [3]. The pathogens of donor-derived and non-donor-derived IFIs are similar and include invasive candidiasis, cryptococcosis, aspergillosis, zygomycosis, and infection from endemic fungi such as histoplasmosis, blastomycosis, and coccidioidomycosis [1, 3, 4].

The main portal of entry for many of these fungi is the lungs, which is problematic specifically in lung transplantation. In the largest compilation of IFIs in SOT, lung transplantation carried the second highest incidence (8.6%) of IFIs, trailing only small bowel transplants [1]. IFIs among patients who underwent lung transplantation (*n* = 248) were reported due to* Aspergillus* (*n* = 109)*, Candida* (*n* = 49), and other molds (*n* = 49) [1]. The predominance of* Aspergillus* over* Candida* was not seen in any of the other solid organ transplant types. Less than 10 cases were found due to zygomycosis, cryptococcosis, pneumocystosis, endemic mycoses, and unspecified yeasts or molds. No infections due to* Sporothrix* were identified in this study (personal communication, Pappas) [1].

We report a case of likely donor-derived primary pulmonary sporotrichosis in a recent lung transplant recipient. To our knowledge this is the first reported case of pulmonary sporotrichosis in a lung transplant recipient and the second reported case of pulmonary sporotrichosis in any solid organ transplant recipient.

## 2. Case Presentation

A 49-year-old male with a history of idiopathic pulmonary fibrosis (IPF) was electively admitted for bilateral single lung transplant. The patient had stable lung function in recent months and had been receiving two liters of oxygen via nasal cannula and 7.5 mg of oral prednisone daily. Upon transplantation, induction immunosuppression consisted of high dose methylprednisolone, tacrolimus, and mycophenolate mofetil. The immediate postoperative course was complicated by a hemothorax due to bronchial artery bleeding four hours after transplantation that necessitated mediastinal exploration and clip ligation. In the first five days after transplantation, this patient remained mechanically ventilated with high oxygen and pressure support as well as nitric oxide. He also required vasopressors for circulatory support. Bronchoscopies during this period showed intact anastomoses and mucopurulent drainage.

Donor bronchial cultures grew* Escherichia coli* and* Streptococcus viridans*. After 5 days of perioperative vancomycin and piperacillin-tazobactam, antibiotics were transitioned to ceftriaxone targeting the donor culture results. The patient continued to require significant ventilator support. Due to grade 3 primary graft dysfunction, high dose steroids were administered from day +10 through day +12 after transplant for presumed rejection and were subsequently tapered while tacrolimus and mycophenolate mofetil were continued.

**Figure 1:**
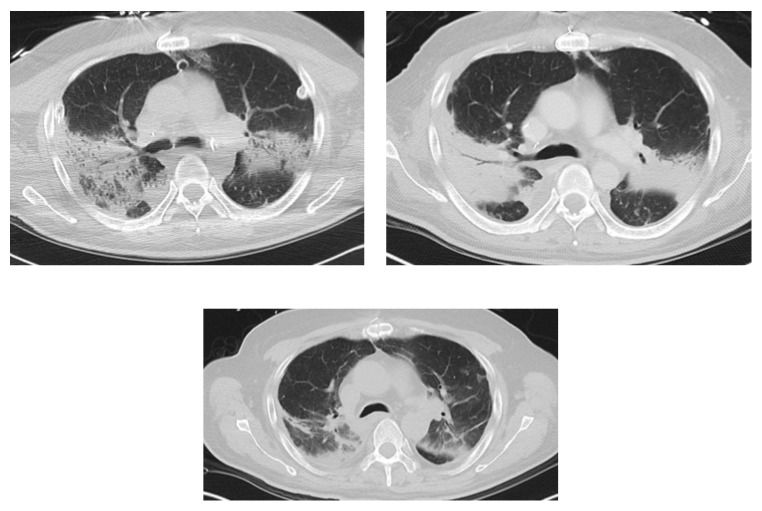
Serial chest CT imaging prior to and after treatment with amphotericin. The first image shows the patient's chest at day +12 after transplantation with significant disease in the dependent areas of the lungs bilaterally that has progressed to dense consolidation by day +25 (3 days prior to diagnosis) as the second figure shows while the third image shows the patient's chest imaging at day +90 with significant improvement.

On day +15 the patient was noted to have a temperature of 102.3°F with an ongoing leukocytosis as high as 25.4 cells/*μ*L. Ceftriaxone was changed back to vancomycin and piperacillin/tazobactam. Mechanical ventilation continued with a fraction of inspired oxygen (FiO2) of 55–60%. Bronchoscopy continued to show mucopurulent secretions, and bronchoalveolar lavage (BAL) cultures grew* Escherichia coli* (sensitive to all tested antibiotics), coagulase negative* Staphylococcus, Enterococcus* spp. (sensitive to all tested antibiotics), and* Candida famata*. Voriconazole was initiated at the time of the reported* Candida* result. Low-grade intermittent fevers up to 100.2°F continued until transplant day +27 with ongoing leukocytosis. Mechanical ventilator support continued with FiO2 maintained at 40–50%. Serial chest CT studies showed increasing extensive airspace interstitial opacities in both lungs (Figure 1).

A BAL fungal culture from day +2 identified* Sporothrix schenckii*; on day +28 after transplant, sensitivity analysis was not completed. At the time of fungus identification amphotericin B lipid formulation was started at 5 mg/kg/day after which the low-grade fevers and leukocytosis resolved. Overall respiratory function stabilized and slowly improved. The patient tolerated the amphotericin product for a total of 30 days with a creatinine of 0.42 mg/dL at the start of therapy and 0.49 mg/dL on the day amphotericin therapy was completed. Itraconazole 200 mg oral solution was initiated three times daily at day 15 of amphotericin therapy (transplant day +43). Itraconazole levels were found to be 0.90 *μ*g/mL at day 14, 0.39 *μ*g/mL at day 34, and 0.78 *μ*g/mL at day 68 of itraconazole therapy, and liver function tests remained normal while on therapy. After stopping amphotericin the patient has remained on itraconazole monotherapy at 200 mg three times daily with a planned course of at least one year. Follow-up CT chest day +89 showed improved bilateral peribronchial consolidation (Figure 1). Subsequently the patient's condition has continued to slowly improve.

Neither the patient nor donor in this case had any known occupation or hobbies that put him/her at risk for sporotrichosis. The donor's death was not traumatic and did not occur outdoors.

## 3. Conclusions

To our knowledge this is the first reported case of* S. schenckii* pulmonary infection in a lung transplant recipient. The organisms was isolated from a BAL specimen on the second day after transplantation but was not identified until day +28. This patient had primary graft dysfunction, ongoing low-grade fevers with leukocytosis, and diffuse pulmonary infiltrates, all of which slowly resolved after initiation of amphotericin. Importantly, successful treatment occurred with amphotericin induction followed by itraconazole maintenance therapy.

*Sporothrix schenckii* is a dimorphic environmental fungus commonly found in decaying material throughout the world [11]. Localized cutaneous skin ulceration or nodules (“Rose Gardener's Disease”) are the most common syndrome, which occurs from direct skin inoculation [11]. Less commonly,* Sporothrix* causes pulmonary disease through either direct inhalation of the fungus or seeding of the lungs from systemic dissemination [11, 12]. The syndrome of pulmonary sporotrichosis has been categorized based on radiographic patterns into cavitary or noncavitary forms [13]. This has significance in that surgery in combination with medical management is often needed to improve outcomes in cavitary sporotrichosis [13].

Primary pulmonary sporotrichosis (inhalational) presents similar to tuberculosis with fatigue, weight loss, and granulomatous pulmonary disease. This form often forms cavities and has a predilection for the upper lobes [13]. Comparatively, noncavitary sporotrichosis most commonly occurs from disseminated infection, is associated with immunocompromised states, and is treated successfully in up to 63% of cases with medical management alone [13]. Notably our patient had diffuse infiltrative lung disease from* Sporothrix schenckii* during primary infection, which we attributed to the immunocompromised state of patient after transplant.

Seven other cases of* Sporothrix schenckii* have been described in SOT recipients (Table 1) [5–9]. Six of the seven cases occurred in renal transplant recipients greater than one year after transplant. Of those six cases, the portal of entry was skin in four cases, inhalation in one (which was pulmonary* Sporothrix*), and presumably the renal collecting system in the last case. Of the four cases that started as cutaneous infections, two progressed to osteomyelitis via local invasion, one had systemic dissemination with meningitis, and one case remained a fixed cutaneous infection.

The 7th case was reported in a study designed to describe the interactions between itraconazole and cyclosporine [10]. This series included 7 fungal infections (5 aspergillosis, 1 coccidiosis, and 1 sporotrichosis) among 7 recipients of thoracic organs (4 heart-lung, 2 heart, and 1 lung). Six of the patients had pulmonary infections and one had a knee infection. Unfortunately the authors do not describe the body site infected by each pathogen [10]. In the two largest compilations of IFI in SOTs, cumulatively representing 1,231 IFIs (1,208 non-donor-derived, 23 donor-derived IFIs), none of these cases were reported to be due to* Sporothrix* [1, 3]. Additionally, one reported case of a possible pulmonary infection due to* Sporothrix cyanescens* (a different organism than* S. schenckii*) in a heart transplant recipient has been reported. In this case, the acquisition of the fungal infection was thought to be zoonotic and was not of certain pathologic significance [14, 15].

The impact of donor-derived fungal infections on SOT has not been fully elucidated and is only now beginning to be systematically studied [16]. Since 2005, the Organ Procurement Transplant Network (OPTN) has required reporting of all unanticipated potential donor-derived transmission events, including malignancies and infections. Since that time, the incidence of reported events continues to increase each year, with 43% more events reported in 2013 (*n* = 284) compared to 2012 (*n* = 198) [16]. Of the 284 events in 2013, 204 were infections with 18% (37/204) representing fungal infections [16]. Among these possible donor-derived fungal infections* Candida* was noted most frequently, with cases of cryptococcosis, histoplasmosis, coccidioidomycosis, and aspergillosis being noted; nine cases were classified as other fungal infections [16]. Lung transplantation had the highest rate of disease transmission from donors, occurring among 80% of exposed lung recipients [16]. Even though only 1.1% of donors were associated with a report of a potential donor-derived transmission event (the incidence of donor-derived fungal infections is estimated to be approximately one percent [2, 16]), when donor-derived infections do occur, they are associated with significant mortality.

To our knowledge this is the first case of likely donor-derived* Sporothrix schenckii* infection. This lung transplant recipient had stable pulmonary function and no known risk factors (aside from IPF) for pulmonary sporotrichosis prior to transplantation. He then received a bilateral single lung transplant from which* Sporothrix schenckii* was isolated on the second day after transplant. Cultures taken from the recipient's native lungs (including fungal cultures) at the time of transplant were negative. Given these factors, we believe that this was likely a donor-derived infection. Other cases of donor-derived IFIs due to environmental molds have occurred in the setting of traumatic events in the donor such as near drowning (*Apophysomyces elegans* [17],* Scedosporium apiospermum* [18]), being discovered face-down in soil (mucormycosis) [19], or receiving thoracostomy tubes in the field (*Scopulariopsis*) [20]. In our case, the donor did not have any clear occupational or hobby-related exposures that we could identify and did not suffer a trauma related death. Nonetheless, the recipient had rapid onset of pulmonary disease attributed to sporotrichosis very early after transplant with primary graft dysfunction, which is characteristic of donor-related infections [3]. The clinical and radiographic response to the initiation of an amphotericin product indicates that* S. schenckii* was a real pathogen in this case.

In conclusion, we report the first case of probable donor-derived* Sporothrix schenckii* infection in a lung transplant recipient, which was successfully treated with amphotericin and itraconazole. The true incidence and impact of donor-derived infections are still being delineated. Use of appropriate microbiologic data is essential in diagnosing these infections, and screening with sputum/bronchial cultures in lung transplant donors and recipients remains instrumental. Physicians should keep in mind the potential for donor-derived fungal infections in the proper clinical setting including rare pulmonary pathogens such as* Sporothrix schenckii.*

## Figures and Tables

**Table 1:** Description of reported cases of *Sporothrix schenckii *infection in solid organ transplant recipients.

Transplanted organ	Timing of infection	Infection location	Geographic location	Therapy
Kidney [5]	4 years after transplant	Cutaneous	Brazil	Amphotericin × 10 days followed by itraconazole

Kidney [5]	Less than one year after transplant	Disseminated	Brazil	Itraconazole initially and then amphotericin after relapse followed by itraconazole

Kidney [6]	Recurrence of a prior infection 7 years after transplant	Cutaneous, relapsed with osteomyelitis	Italy	Fluconazole initially and at relapse

Kidney [7]	<1 year after transplant	Pulmonary	India	Not specified

Kidney [8]	1-2 years after transplant	Renal	India	Died prior to treatment of unrelated cause

Kidney [9]	4 years after transplant	Disseminated, CNS, and bone	USA	Amphotericin

Thoracic [10] (unclear if lung, heart, or both)	Unclear	Pulmonary or articular	USA	Itraconazole
